# Estrogen Enhances the Cell Viability and Motility of Breast Cancer Cells through the ERα-ΔNp63-Integrin β4 Signaling Pathway

**DOI:** 10.1371/journal.pone.0148301

**Published:** 2016-02-04

**Authors:** Jar-Yi Ho, Fung-Wei Chang, Fong Shung Huang, Jui-Ming Liu, Yueh-Ping Liu, Shu-Pin Chen, Yung-Liang Liu, Kuan-Chen Cheng, Cheng-Ping Yu, Ren-Jun Hsu

**Affiliations:** 1 Department of Pathology, and Graduate Institute of Pathology and Parasitology, Tri-Service General Hospital, National Defense Medical Center, Taipei, Taiwan; 2 Department of Obstetrics & Gynecology, Tri-Service General Hospital, National Defense Medical Center, Taipei, Taiwan; 3 Department of Integrated Diagnostics & Therapeutics, National Taiwan University Hospital, Taipei, Taiwan; 4 Division of Urology, Department of Surgery, Taoyuan General Hospital, Ministry of Health and Welfare, Taoyuan, Taiwan; 5 Department of Emergency Medicine national Taiwan University Hospital, Taipei, Taiwan; 6 Division of Medical Genetics, Department of Pediatrics, Chang Gung Children's Hospital, Taoyuan, Taiwan; 7 Institute of Biotechnology, National Taiwan University, No. 1, Sec. 4, Roosevelt Road, Taipei, Taiwan; 8 Biobank Management Center of Tri-Service General Hospital, National Defense Medical Center, Taipei, Taiwan; University of California at Davis, UNITED STATES

## Abstract

Estrogen induces ERα-positive breast cancer aggressiveness via the promotion of cell proliferation and survival, the epithelial-mesenchymal transition, and stem-like properties. Integrin β4 signaling has been implicated in estrogen/ERα-induced tumorigenicity and anti-apoptosis; however, this signaling cascade poorly understood. ΔNp63, an N-terminally truncated isoform of the p63 transcription factor, functions as a transcription factor of integrinβ4 and therefore regulates cellular adhesion and survival. Therefore, the aim of the present study was to investigate the estrogen-induced interaction between ERα, ΔNp63 and integrin β4 in breast cancer cells. In ERα-positive MCF-7 cells, estrogen activated ERα transcription, which induced ΔNp63 expression. And ΔNp63 subsequently induced integrin β4 expression, which resulted in AKT phosphorylation and enhanced cell viability and motility. Conversely, there was no inductive effect of estrogen on ΔNp63-integrinβ4-AKT signaling or on cell viability and motility in ERα-negative MDA-MB-231 cells. ΔNp63 knockdown abolishes these estrogen-induced effects and reduces cell viability and motility in MCF-7 cells. Nevertheless, ΔNp63 knockdown also inhibited cell migration in MDA-MB-231 cells through reducing integrin β4 expression and AKT phosphorylation. In conclusion, estrogen enhances ERα-positive breast cancer cell viability and motility through activating the ERα-ΔNp63-integrin β4 signaling pathway to induce AKT phosphorylated activation. Those findings should be useful to elucidate the crosstalk between estrogen/ER signaling and ΔNp63 signaling and provide novel insights into the effects of estrogen on breast cancer progression.

## Introduction

Overexpression of the estrogen receptor alpha (ERα) is observed in approximately 70% of all breast cancer patients, and most breast cancer patients initially respond to anti-estrogen therapy. Approximately 20% to 40% of patients with breast cancer eventually relapse in distant organs (i.e., metastasis), which remain undetectable for years after primary tumor diagnosis, and this phenomenon is commonly observed in ERα-positive breast cancer. Multiple ERα mechanisms have been proposed to explain how cancer cells survive and relapse [1].

Integrin β4 is a cellular adhesion molecule that heterodimerizes with integrin α6 and functions as a receptor for laminins in the extracellular matrix. Integrin β4 pairs only with integrin α6, thus making integrin β4 expression predictive of the integrin α6β4 heterodimer [2]. Integrin α6β4 is predominantly expressed in epithelial cells and is localized to the basal surface adjacent to the basement membrane to nucleate the formation of hemidesmosomes [2,3]. Integrin α6β4 dissociation from hemidesmosomes is involved in multiple signaling pathways during carcinoma progression [2, 4]. Dissociated integrin β4 directly binds to laminin to activate phosphoinositide 3-OH kinase (PI3K)/AKT signaling [5], which subsequently promotes cell proliferation and survival [6] and cell invasiveness [3, 5]. Overexpression of integrin β4 has been associated with the aggressive behavior and poor prognosis of breast cancer and other cancer types [7]. Loss of integrin β4 signaling inhibits mammary tumor onset and inhibits tumor invasion and metastasis to the lungs [8]. ERα signaling has been shown to indirectly participate in the activation of integrin β4 signaling [9, 10]. Loss of integrin β4 reduced tumorigenicity in the ERα-positive breast cancer cell line MCF-7 and even induced apoptosis under estrogen deprivation [11]. However, it remains unknown how ERα activates integrin β4 signaling.

The *TP63* gene belongs to the *TP53* gene family that also includes *TP73*. Similar to other members of the p53 family, the *TP63* gene is expressed as multiple isoforms according distinct promoter usage. TAp63 is a full-length form possessing a transactivation (TA) domain that is encoded from a transcript using promoter-1, and ΔNp63 is an amino-deleted isoform with a truncated N-terminus (ΔN) that is encoded from a transcript using promoter-2 [12, 13]. TAp63 exerts tumor suppressor role that regulates genes involved in cell cycle inhibition [14, 15] and apoptosis [14, 16] through induction of p53-regulated genes [17] or non-p53-related genes [18, 19]. ΔNp63 exerts oncogenic properties through the transactivation of genes involved in the cell cycle [20, 21], anti-apoptosis [22], cell migration/invasion [23, 24], angiogenesis [25] and cancer cell stemness [26–28]. TAp63 and ΔNp63 exert mutual inhibitory effects. TAp63 activates the Notch signaling pathway to inhibit *ΔNp63* gene expression [29, 30]. Conversely, ΔNp63 acts as a dominant-negative inhibitor of TAp63 [31] and inhibits TAp63 induction of p53-related downstream genes [13, 32], thereby promoting the expression of anti-apoptotic genes. ΔNp63 protein is predominantly overexpressed in breast cancer [33] and several other cancer types [34–36], and higher expression of ΔNp63 is associated with a poorer prognosis [33, 37, 38]. The *TP63* gene is necessary for epithelial development; that is, a full knockout of *TP63* is lethal, owing to the absence of the epidermis [39, 40], which results in severe dehydration and the absence of epidermal appendages, such as hair, sebaceous glands, limbs, skin and other organs [39–41]. Besides, the *TP63* gene is rarely mutated in human cancers, indicating that p63is not a canonical tumor suppressor. Overexpression of ΔNp63 induces more tumorigenicity and more cancer stem cell phenotypes in MCF7 cells [42]. Moreover, ΔNp63 has been reported to function as a transcription factor of integrin β4, thereby regulating cellular adhesion and survival [43]. Given the effects of ERα on integrin β4, we hypothesized that estrogen activates the ERα-ΔNp63-integrin β4 axis in ERα-positive MCF-7 breast cancer cells and induces cell viability and cell motility.

## Materials and Methods

### Cell lines and transfection

MCF-7 (ERα-positive) and MDA-MB-231 (ERα-negative) breast cancer cell lines were purchased from the Bioresource Collection and Research Center and maintained in Dulbecco’s Modified Eagle Medium (DMEM) containing 10% fetal bovine serum (FBS) at 37°C in a5% CO_2_ atmosphere.

For transfection, 1 × 10^6^MCF-7 and MDA-MB-231 cells were transfected with 60 pmol small interfering RNAs (siRNAs) of ERα (siESR1), ΔNp63 (siΔNp63) or scrambled siRNA (scr) for 48 h. The transfection was performed with Lipofectamine 2000 Reagent (Invitrogen, Carlsbad, CA, USA) according to the manufacturer's instruction. The transfected cells were collected for subsequent mRNA or protein assays.

### Cell viability detection

Cell viability was evaluated with MTT assay (MTT reagent, Bio Basic, Markham, Ontario, Canada). After 2 × 10^3^ cells were seeded in each well of a 96-well microplate and overnight culture, fresh medium (10% FBS/DMEM) containing different concentrations of estrogen (17-β-estradiol, E2758, Sigma-Aldrich, Saint-Louis, MO, USA) or 0.1% ethanol solvent control was replaced and maintained for four consecutive days. The absorbance at 570 nm (A570) was detected with a Multiska Microplate Photometer (Thermo Fisher Scientific Inc, Waltham, MA, USA). The cell growth curve was plotted from the average A570 values of six repeated measurements.

Cell viability was also determined using a trypan blue exclusion assay [44]. In brief, 2 ×10^3^ cells were seeded and treated as MTT assay. After trypsinization and washing, harvested cell pellet was resuspended with 1 mL phosphate-buffered saline (PBS, pH 7.2), and mixed with 0.1 mL 0.4% solution of trypan blue (Thermo Fisher Scientific Inc, Waltham, MA,USA) for 3 min, and then a drop of the mixture was loaded onto a hemacytometer to count the number of blue stained and total cells immediately under a microscope at l00× magnification. Cells were considered non-viable if they took up trypan blue. The percentage of viable cells was calculated as follow: [1.00 –(number of blue cells ÷ number of total cells)] × 100. The cell growth curve was plotted from the average viable cell percentage from six repeated measurements.

### Migration assays

For wound-healing experiments, cells were seeded in a 6cm culture dishes to reach >90% confluence. After attachment, cells were scraped with a p200 tip at time 0 to draw a cell-free line (approximately 1 mm in width). After removing the original medium and washing with PBS, fresh medium containing 10nM estrogen or 0.1% ethanol was added. The condition of wound healing was observed and photographed under a microscope at 0, 12, 24, and 48h. Transwell migration assay were assessed with 8μm inserts (BD Biosciences). 1×10^4^ cells were suspended in 100μl fresh medium and loaded into upper insert, and lower chambers were filled with 500μl fresh medium. The chambers were incubated at 37°C in a 5% CO_2_ atmosphere for 24hr. Cells were then fixed with 500μl methanol for 15min and wiped the inner surface of the upper inserts by using cotton swabs to remove the un-migrated cells. After washed with PBS and stained with hematoxylin, and the transwell membranes were torn and kept in slides. The wound-healed distances were measured from each picture and the stained migrated cells on each tranwell membrane were counted from five random fields using Image J software (NIH, USA). Each experiment was repeated at least three times independently.

### Western blotting

Crude cell lysates were harvested using RIPA lysis buffer (Merck Millipore, Billerica, MA, USA)f ollowing the manufacturer’s instructions. Proteins were separated by 10% SDS-polyacrylamide gel electrophoresis and transferred to a polyvinylidene difluoride membrane. Non-specific protein binding was prevented with the addition of blocking buffer (5% milk, 20mM Tris-HCl [pH 7.4], 150mM NaCl, and 0.1% Tween-20) and blotted with specific primary antibodies in the blocking buffer at 25°C for 90min, followed by incubating with the secondary antibody for 60 min. Proteins were visualized using horseradish peroxidase -conjugated secondary antibodies (Jackson ImmunoResearch, West Grove, PA, USA) and enhanced chemiluminescent reagent (Pierce, Thermo Fisher Scientific Inc, Waltham, MA,USA) for western blots. Specific primary antibodies included rabbit-anti-human ERα (Abcam, ab32063, clone E115), rabbit-anti-human ΔNp63 (Calbiochem, 5–17), rabbit-anit-human TAp63 (BioLegend, 618902) and mouse-anti-human β-actin (Thermo Scientific, MA5-15739, clone BA3R).

### Real-time reverse transcriptase-polymerase chain reaction (real-time RT-PCR)

Total cellular RNA was isolated from subconfluent cells cultured in a 6cm dish using TRIzol Reagent (Invitrogen, Carlsbad, CA, USA) following the manufacturer’s instructions. The RNA concentration was determined using a NanoDrop spectrophotometer (Thermo Fisher Scientific Inc, Waltham, MA, USA). Reverse transcription was performed with 2 μg total RNA using SuperScript III Reverse Transcriptase (Invitrogen, Carlsbad, CA, USA). Real-Time PCR was performed with SYBR Green qPCR Master Mix (2X) (Fermentas, Thermo Fisher Scientific Inc, Waltham, MA, USA) and was measured using real-time PCR systems (StepOne, Applied Biosystems Carlsbad, CA, USA). Cycle threshold values are reported relative to the levels of *GAPDH* mRNA.

### Construction of ΔNp63 and ITGB4 (integrin β4) promoter luciferase plasmids

The PCR reactions were carried out with sequence specific primer pairs; these primers were all designed to contain a XhoI site and a HindIII site for subsequent cloning into the luciferase reporter vector pGL4.21, which is a basic vector with no promoter (Promega, Madison, WI, USA). The 3,277-bp fragment containing the human *ΔNp63* promoter was designed from NC 000003.12 and amplified from human genomic DNA using the primers 5’-CTCGAGAAGGCCTTTCACGCTGTGTCTGAA -3’ and 5’-AAGCTTTCCATCAACTTCCACCTGGACTCCT -3’. The fragment was inserted into pGL4.21 via the XhoI and HindIII sites to obtain vector pGL4.21-ΔNp63-Luc. Similarly, the 2,951-bp fragment containing the human *ITGB4* promoter was designed from NC_000017.11 and amplified from human genomic DNA using primers 5’-CTCGAGCCTAAGGCTGCAGAGATAGTGGGGT -3’ and 5’-AAGCTTTCAGGGAGGGGAAGGGGAGACAAAAGAAGCTT-3’. The fragment was also inserted into pGL4.21 via XhoI and HindIII sites to obtain pGL4.21-ITGB4-Luc. Proper insertion was verified by direct DNA sequencing.

### Luciferase assay

Transient transfection of luciferase reporter plasmids was also performed using Lipofectamine 2000. The cells were seeded in 12-well tissue culture plates at 2 × 10^5^ per well and co-transfected with 0.5μg of luciferase reporter plasmids (pGL4.21-ΔNp63-Luc or pGL4.21-ITGB4-Luc) and 0.2μg pGL4.74[hRluc/TK] Vector (Promega, Madison, WI, USA), an internal control reporter. To analyze ΔNp63 promoter activity in response to estrogen, cells were treated under 10nM estrogen or 0.1% ethanol for 2 h. And to analyze ITGB4 promoter activity, cells were co-transfected with siΔNp63 or scr control for 48h and then treated with 10mM estrogen or 0.1% ethanol for 2 h. After washing with PBS, cells were lysed in 150 μL of 1 × reporter lysis buffer (Promega, Madison, WI, USA) and lysates were used directly for a firefly luciferase activity assay using Firefly and Renilla Dual-Reporter Luciferase Assay Systems (E1910, Promega, Madison, WI, USA) according to the manufacturer’s instructions. Renilla luciferase activity was subsequently detected with the same lysates and kit to standardize transcription efficiency. All experiments were performed at least six times, and average relative luciferase activity was obtained for plotting the bar-charts.

### Chromatin immunoprecipitation (ChIP)

A chromatin immunoprecipitation assay was performed in MCF-7 cells using a ChIP-IT Express Chromatin Immunoprecipitation Kit (Active Motif, Carlsbad, CA, USA) according to the manufacturer’s instructions. Collected and precleaned cell lysate was immunoprecipitated with 2μg of anti-ERα (Abcam, clone E115, ab32063) or 2μg of non-immunized rabbit IgG (Abcam, ab171870). After immune complexes elution, heat crosslink, and treatment with RNase A and proteinase K, the DNA was recovered using a Gel/PCR DNA Fragments Extraction Kit DF100 (Geneaid, Taiwan). After purification, PCR was used to analyze immunoprecipitated DNA with the following primers:5’- GTTAGCATTTTGTTATTTCTGGAAAGCAT -3’ (forward) and 5’- CAAGGATTTTACGTCAATGTCCCAA -3’ (reverse). Those primers were designed to flank the most conserved estrogen response element (ERE) between -2,858bp to -2,839bp from the translation start site (ATG) within the *ΔNp63* promoter which was predicted with TFBIND [45]. And 1μg purified DNA was subjected to 30 cycles of PCR amplification in a volume of 25μL (denaturing at 95°C for 1 min, annealing at 60°C for 1 min, extension at 72°C for 1 min, and a final extension at 72°C for 5 min). The 234-bp PCR products was separated by agarose gel electrophoresis and visualized by ethidium bromide staining.

### Statistical Analysis

Results of real-time PCR, western blot, MTT and trypan blue exclusion assay, reporter assays, and migration assays were recorded as continuous data and analyzed with Student’s *t*-test. Statistical analyses involved the use of SPSS 16.0 and Microsoft Excel 2010. All statistical tests and *P*values were two sided. *P*< 0.05 was considered to be statistically significant.

## Results

### Effects of estrogen on the cell viability

To determine the optimal estrogen concentration for subsequent experiments, we compared the effect of a series of estrogen concentrations on the cell viability using MTT and trypan blue exclusion assays (S1 Fig). The cell viability of the MCF-7 cells was markedly better under 10nM estrogen treatment than under 0.1% ethanol treatment, but the cell viability of the MDA-MB-231 cells was not modified by different concentrations of estrogen. Therefore, a concentration of 10nM estrogen was used in subsequent experiments

### Effects of estrogen on ΔNp63 and TAp63 expression

**Fig 1:**
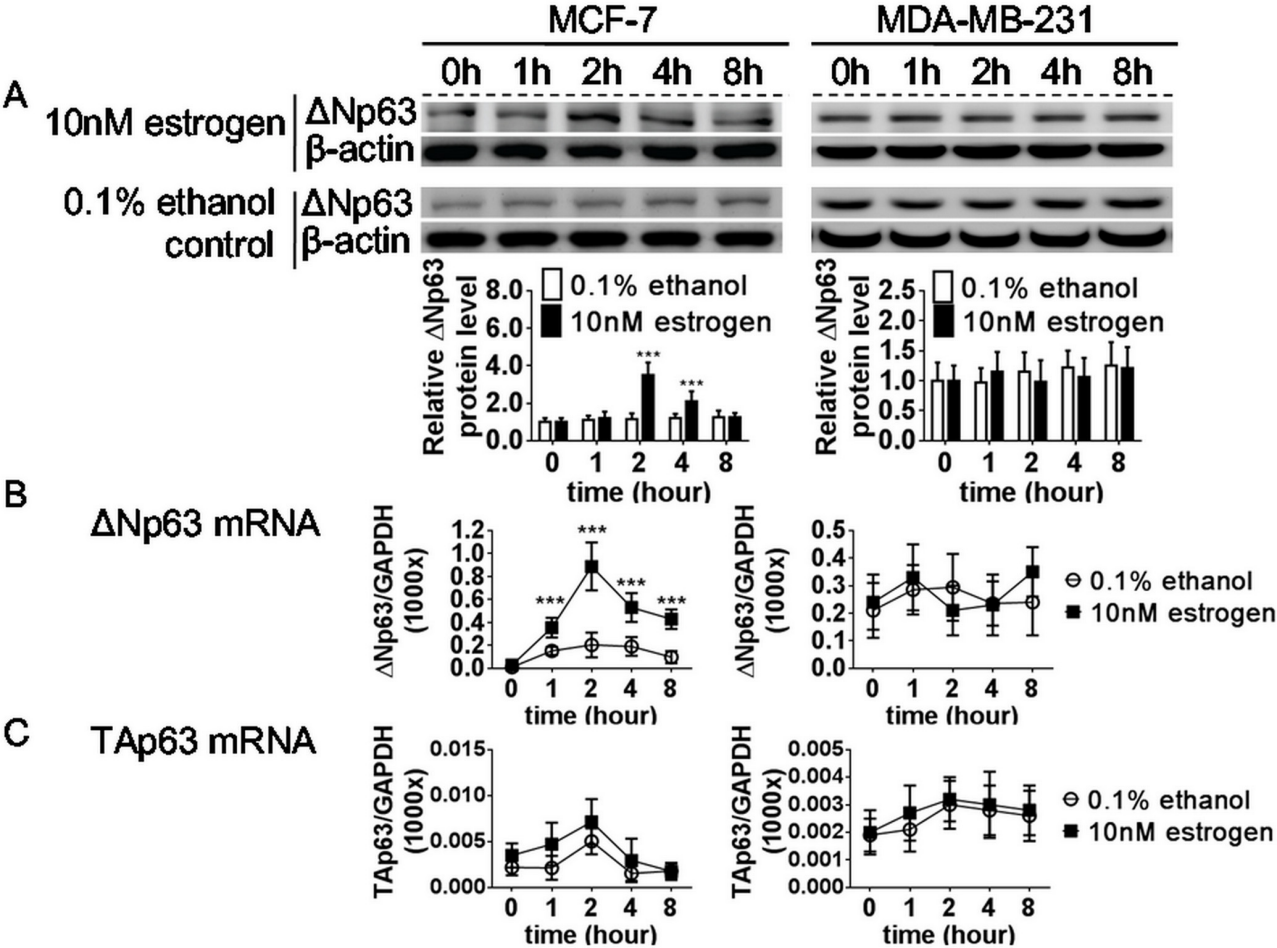
Effects of estrogen on ΔNp63 and TAp63 expression in MCF-7 and MDA-MB-231 cells. MCF-7 and MDA-MB-231 cells were cultured in 10% FBS/DMEM with 10nM estrogen or 0.1% ethanol for 0, 1, 2, 4, and 8 h, and protein and mRNA expression was detected by (A) western blotting and real-time RT-PCR for (B) ΔNp63 and (C) TAp63. In MCF-7, ΔNp63 expression peaked at 2 h after estrogen treatment at both the protein and mRNA levels, and estrogen treatment did not significantly affect TAp63 protein and mRNA expression levels. In MDA-MB-231, estrogen treatment did not affect ΔNp63 and TAp63 expression in both protein and mRNA levels. All data are the mean ± SD of triplicate experiments.

After estrogen treatment, the protein expression of ΔNp63 was fluctuated over time in the MCF-7 cells and peaked at 2 h. And the protein expression of ΔNp63 was maintained at similarly lower levels over time in the 0.1% ethanol-treated control group (Fig 1A). In contrast, the protein expression of ΔNp63 was also similar over time in MDA-MB-231 cells regardless of the presence or absence of estrogen (Fig 1A). These results suggest that estrogen induced the expression of the *ΔNp63* in MCF-7 cells.

The mRNA expression level of *ΔNp63* was also significantly induced after estrogen treatment and peaked at 2 h (Fig 1B); however, the mRNA expression of *TAp63* was much lower than *ΔNp63* and non-significantly fluctuated, regardless of the presence or absence of estrogen, in the MCF-7 cells (Fig 1C). The mRNA expression of both *ΔNp63* and *TAp63* were similar over time in MDA-MB-231 cells regardless of the presence or absence of estrogen (Fig 1C).

### Estrogen affects ΔNp63 expression through ERα-dependent signaling

**Fig 2:**
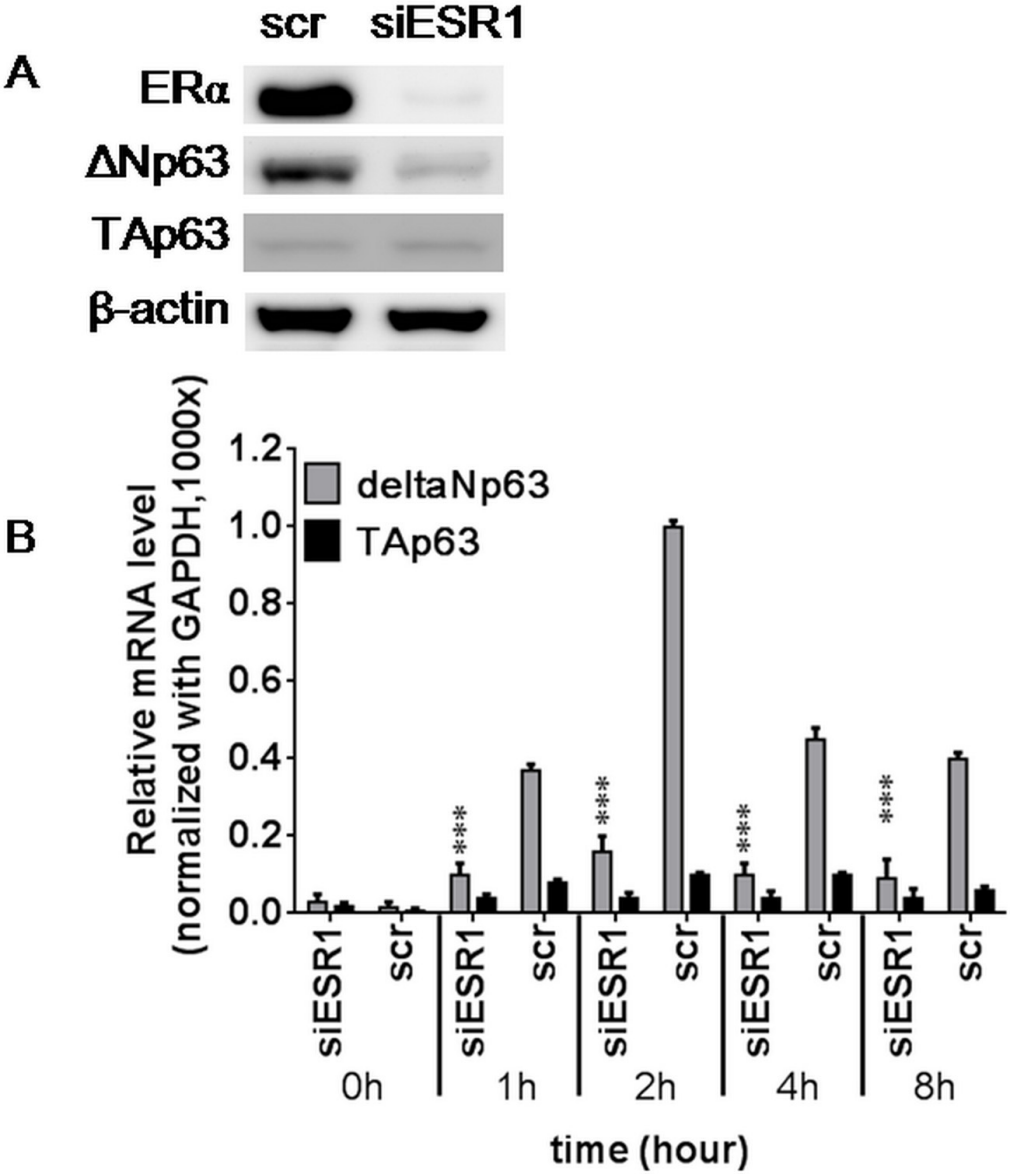
Estrogen receptor α knockdown reduced ΔNp63 expression. ERα-short interfering RNA (siESR1) and scrambled control siRNA (scr) were transfected into MCF-7 cells for 48 h, and then cells were treated with 10nM estrogen or 0.1% ethanol for 0, 1, 2, 4, and 8 h. (A) After ERα knockdown, the protein levels of ERα and ΔNp63 were dramatically reduced, but TAp63 showed an extremely low expression level, regardless of siESR1 transfection. (B) After ERα knockdown, the mRNA expression levels of *ΔNp63* and *TAp63* were detected by real-time RT-PCR, and *ΔNp63* mRNA was decreased significantly compared with the control group. However, *TAp63* mRNA was not affected by the presence or absence of siESR1. All data are the mean ± SD of triplicate experiments.

Combined treatment with 10nM estrogen and ERα-siRNA (siESR1) reduced ERα and ΔNp63 protein expression in siESR1-transfected MCF-7 cells compared with the scrambled siRNA-transfected controls, but TAp63 protein was expressed at a barely detectable level, regardless of the presence or absence of siESR1 (Fig 2A). The mRNA expression of *ΔNp63* was significantly reduced in the siESR1 transfected MCF-7 cells, whereas *TAp63* mRNA remained low expression levels, regardless of the presence or absence siESR1 (Fig 2B). These results suggest that estrogen induced ΔNp63 mRNA and protein expression through an ERα dependent signaling pathway. However, *TAp63* mRNA expression was not affected by estrogen in the MCF-7 cells, regardless of the presence or absence of siESR1.

### Estrogen activates the ΔNp63-integrin β4 axis

**Fig 3:**
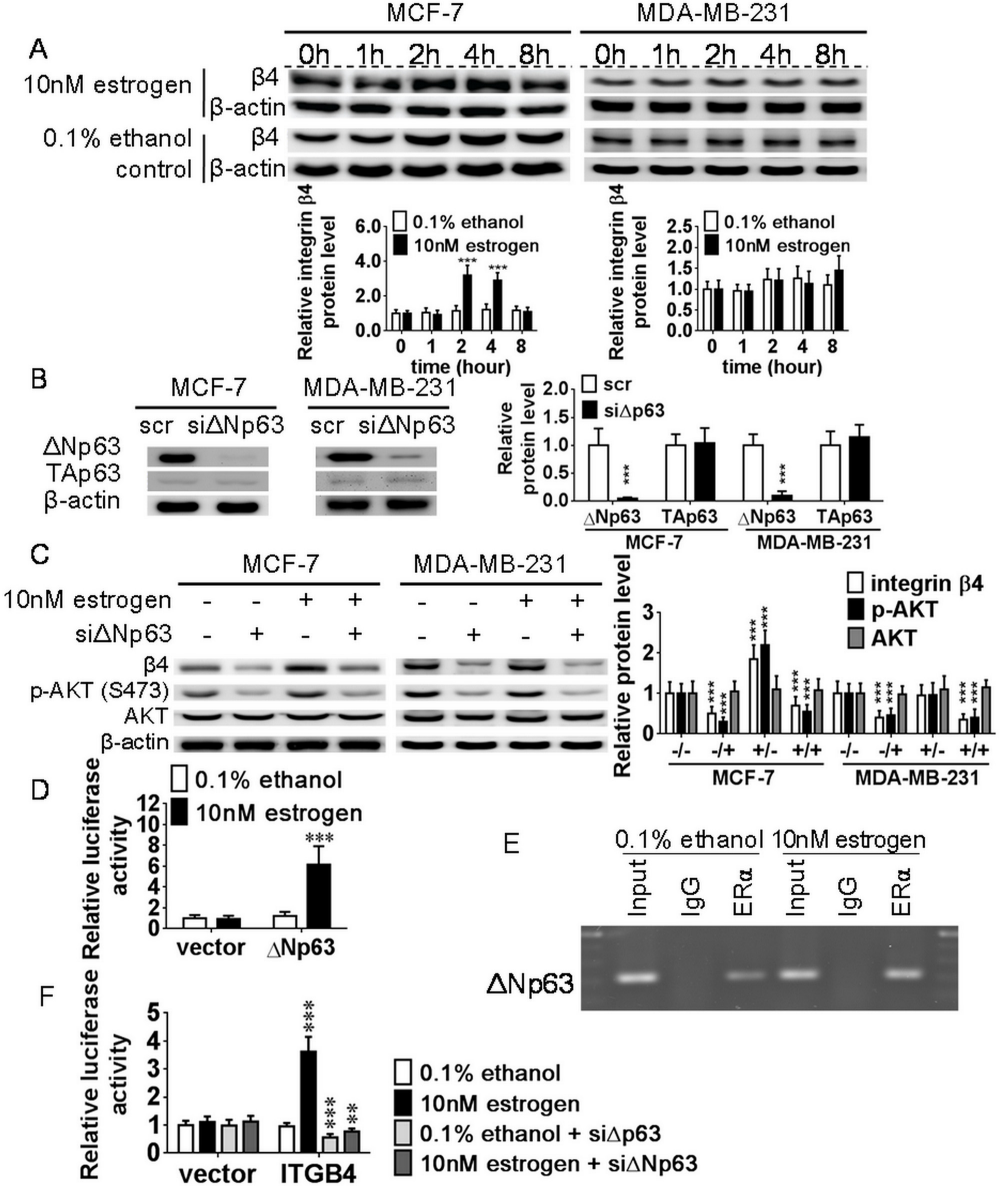
The combined effects of estrogen and ΔNp63 knockdown on integrin β4 expression and AKT activation. (A) After MCF-7 and MDA-MB-231 cells were treated with 10nM estrogen or 0.1% ethanol for 0, 1, 2, 4, and 8 h, integrin β4 protein was detected by western blotting. After estrogen treatment, integrin β4 peaked at 2 h in MCF-7 cells but revealed similar levels among 5 time points in MDA-MB-231 cells. (B) After siΔNp63 or scrambled siRNA were transfected into MCF-7 and MDA-MB-231 cells for 48 h, siΔNp63 specifically inhibited the protein expression of ΔNp63 but not TAp63. (C) After siΔNp63 or scrambled siRNA were transfected into MCF-7 and MDA-MB-231 cells for 48 h, cells were co-treated with 10nM estrogen or 0.1% ethanol for 2 h. In MCF-7 cells, estrogen induced integrin β4 expression and AKT activation, whereas siΔNp63 reduced integrin β4 expression and AKT activation. In MDA-MB-231 cells, estrogen conferred no inductive effect on integrin β4 protein level, but siΔNp63 also reduced integrin β4 expression and AKT activation. Statistical analysis was performed in four groups, 0.1% ethanol with scrambled siRNA (-/-), 0.1% ethanol with siΔNp63 (-/+), 10nM estrogen with scrambled siRNA (+/-), and 10nM estrogen with siΔNp63 (+/+). (D) ERα transcriptional activation of the *ΔNp63* promoter was evaluated with a luciferase reporter assay using the full-length *ΔNp63* promoter. In MCF-7 cells, estrogen treatment induced higher luciferase activity than 0.1% ethanol treatment, but no difference was observed in the empty vector-transfected cells, regardless of treatment with 10nM estrogen or 0.1% ethanol. (E)A ChIP assay was performed to evaluate whether ERα directly binds to the most conserved ERE within the *ΔNp63* promoter. In estrogen-treated MCF-7 cells, the 234-bp PCR product of an ERE-containing fragment was more observable than that of the 0.1% ethanol-treated control group, and no PCR product was observed in the non-immunized rabbit IgG control. (F) ΔNp63 transcriptional activation for the *ITGB4* promoter was evaluated with a luciferase reporter assay using the full-length *ITGB4* promoter. In MCF-7 cells, estrogen treatment induced higher luciferase activity than 0.1% ethanol treatment, whereas siΔNp63 reduced luciferase activity under both 10nM estrogen and 0.1% ethanol treatment conditions. There was no difference observed in the empty vector-transfected cells, regardless treatment with 10nM estrogen or 0.1% ethanol. All data are the mean ± SD of triplicate experiments.

The gene expression of integrin β4 increased in the estrogen treated MCF-7 cells and peaked at 2 h, whereas the protein expression of integrin β4 was maintained at similarly lower levels over time in the 0.1% ethanol-treated control group. Besides, estrogen did not affect the integrin β4 protein expression in MDA-MB-231 cells and revealed similar integrin β4 expression pattern in the 0.1% ethanol-treated control group (Fig 3A). This result suggests that estrogen induces integrin β4 expression in ERα-positive cells. To determine whether ΔNp63 participated in the effect of estrogen-induced integrin β4 expression, MCF-7 and MDA-MB-231 cells were transfected with siΔNp63 and treated with estrogen or 0.1% ethanol for 2 h before harvest. SiΔNp63 transfection specifically reduced ΔNp63 protein levels without affection of TAp63 protein levels in both cell lines (Fig 3B). In MCF-7 cells, integrin β4 and active p-AKT (S473) were induced under 10nM estrogen treatment, but they were reduced under siΔNp63 treatment, regardless of co-treatment with 10nM estrogen or not. In MDA-MB-231 cells, siΔNp63 transfection also reduced integrin β4 expression and AKT phosphorylation irrespective of treatment with 10nM estrogen or 0.1% ethanol (Fig 3C). The results showed that ΔNp63 modulated the effects of estrogen-ERα-induced integrin β4 expression and downstream AKT phosphorylation.

To investigate whether ERα directly transactivates the *ΔNp63* promoter, a full-length *ΔNp63* promoter was constructed into the pGL4.21 luciferase reporter plasmid and introduced to the luciferase reporter assay in MCF-7 cells. Estrogen treatment induced higher luciferase activity than 0.1% ethanol treatment; however, no difference was observed in the empty vector-transfected cells, irrespective of treatment with 10nM estrogen or 0.1% ethanol (Fig 3D). Moreover, the ChIP assay was designed for the most conserved ERE within the *ΔNp63* promoter to evaluate whether ERα directly binds to the *ΔNp63* promoter. After estrogen treatment, the PCR product of the fragment flanked ERE within the *ΔNp63* promoter was more observable than that of the 0.1% ethanol-treated control group. Furthermore, no PCR product was observed in non-immunized rabbit IgG control, which suggests that the ERα antibody specifically binds to ERE within the *ΔNp63* promoter (Fig 3E).

The full-length *ITGB4* promoter was also inserted into the pGL4.21 luciferase reporter plasmid to evaluate whether ΔNp63 directly transactivates the *ITGB4* promoter. Estrogen treatment induced higher luciferase activity than 0.1% ethanol treatment; however, luciferase activity was inhibited in the siΔNp63-transfected MCF-7 cells, regardless of presence or absence of estrogen. There was no difference in the empty vector-transfected cells despite co-treatment with 10nM estrogen or siΔNp63 transfection (Fig 3F). In summary, the reporter assays and ChIP assay impliacte that an estrogen-dependent ERα-ΔNp63-integrin β4 signaling pathway promoted the cell viability and motility of ERα-positive breast cancer cells.

### Effects of estrogen and ΔNp63 expression on cell migration

**Fig 4:**
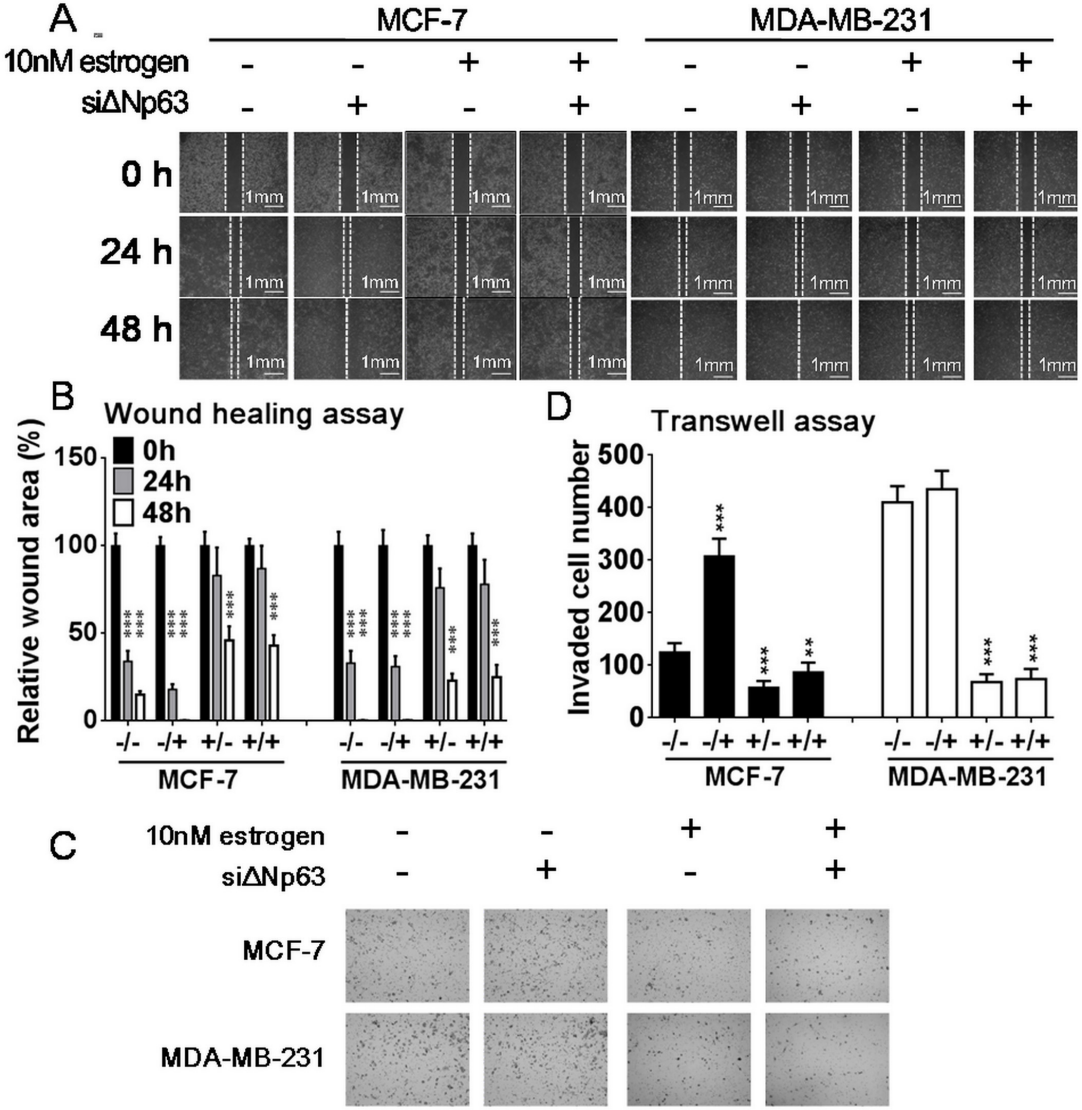
The combined effects of estrogen and ΔNp63 knockdown on cell motility. (A, B) After siΔNp63 or scrambled siRNA transfected into MCF-7 and MDA-MB-231 cells for 48 h, cells were co-treated with 10nM estrogen or 0.1% ethanol and then subjected to a wound-healing assay. Wound widths were recorded at 0, 24, and 48 h. Compared with the scrambled siRNA and 0.1% ethanol co-treated controls, the wounds of MCF-7cells co-treated with scrambled siRNA and 10nM estrogen were completely healed, but siΔNp63 reduced MCF-7 cell healing, regardless of the presence or absence of estrogen. In MDA-MB-231, wounds were completely healed at 48 h irrespective of treatment with 10nM estrogen or 0.1% ethanol, and siΔNp63 also reduced MDA-MB-231 cell healing regardless of the presence or absence estrogen. Statistical analysis was performed in four groups, 0.1% ethanol with scrambled siRNA (-/-), 0.1% ethanol with siΔNp63 (-/+), 10nM estrogen with scrambled siRNA (+/-), and 10nM estrogen with siΔNp63 (+/+). (C, D) After the transfected cells were co-treated with 10nM estrogen or 0.1% ethanol for 24 h, similar results were observed in a transwell assay. In MCF-7 cells, estrogen treatment induced the highest cell migratory effect, but siΔNp63 reduced cell migratory ability, regardless of the presence or absence of estrogen. And in MDA-MB-231 cells, estrogen treatment conferred no inductive effect on cell migration, and siΔNp63 also reduced cell migratory ability, regardless of the presence or absence of estrogen. All data are the mean ± SD of triplicate experiments.

**Fig 5:**
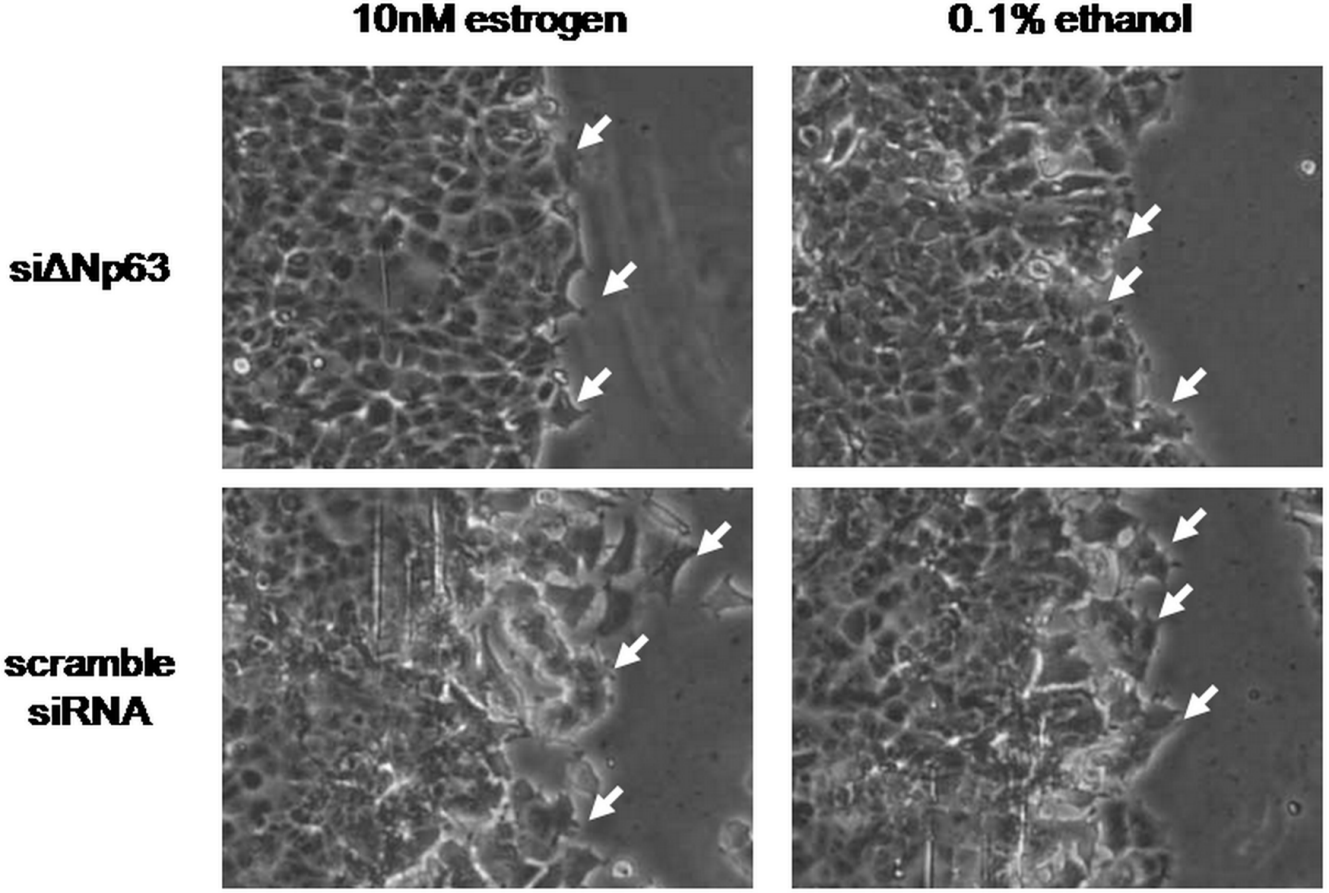
Pseudopodium patterns of co-treatment of estrogen and ΔNp63 knockdown. After siΔNp63 or scrambled siRNA was transfected into MCF-7 cells for 48 h, the cells were co-treated with 10nM estrogen or 0.1% ethanol. A wound-healing assay was conducted to compare the pseudopodium patterns at 24 h. Only the cells treated with 10nM estrogen showed protruding pseudopodia, and siΔNp63 reduced the pseudopodium patterns, regardless of the presence or absence of estrogen.

Wound-healing and transwell assays were used to evaluate the effects of estrogen treatment and/or ΔNp63 knockdownon cell migration of MCF-7 and MDA-MB-231 cells. In scrambled siRNA-transfected MCF-7 cells, wound width was apparently narrower under estrogen treatment than under 0.1% ethanol treatment. After ΔNp63 knockdown, wound width was wider than in the scrambled siRNA transfected controls, with or without estrogen treatment, and this phenomenon was not rescued by co-treatment with 10nM estrogen and siΔNp63 (Fig 4A and 4B). Similar results were also observed in the transwell assay; that is, estrogen induced the migration of more cells compared with 0.1% ethanol treatment in the scrambled siRNA-transfected MCF-7 cells, but fewer migrated cells were observed in the ΔNp63-knockdown MCF-7 cells, regardless of the presence or absence estrogen (Fig 4C and 4D). In MDA-MB-231 cells, estrogen did not induce higher cell motility but siΔNp63 inhibited cell migration regardless of the presence or absence estrogen (Fig 4A–4D). Hence, we speculate that estrogen activated ΔNp63-integrin β-AKT signaling to enhance cell mobility. Furthermore, the lamellipodia were more apparent in the estrogen treated MCF-7 cells compared with the 0.1% ethanol treated controls; however, lamellipodia were reduced in the ΔNp63-knockdown cells (Fig 5). These results implicate that ΔNp63 knockdown reduces the estrogen-induced cell migration of MCF-7 cells.

## Discussion

### 1. Estrogen receptor alpha and ΔNp63 induced similar signaling pathways to promote breast cancer cell viability, motility and stemness

**Fig 6:**
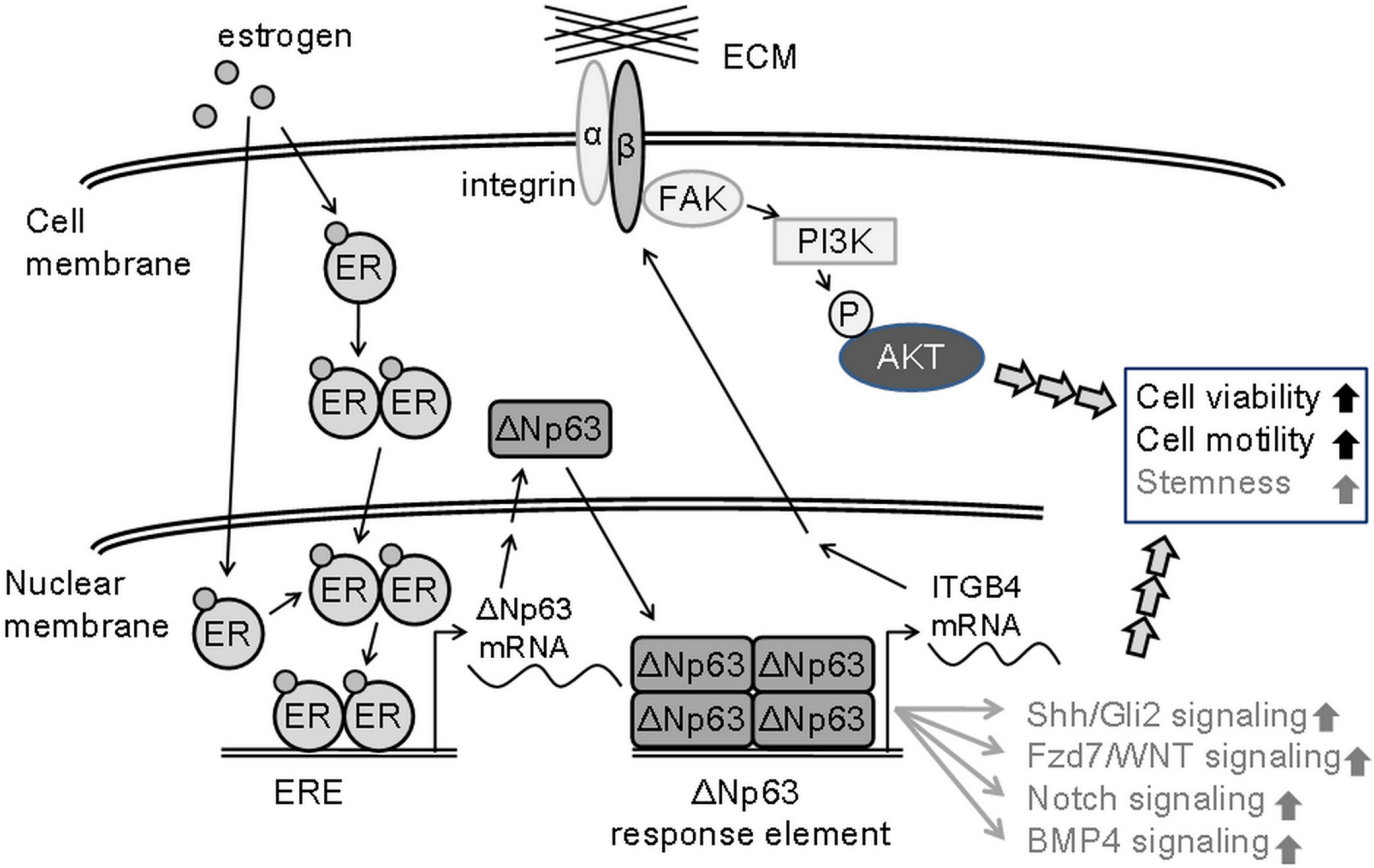
Schematic diagram of ERα-ΔNp63-integrinβ4 axis. ERα-ΔNp63-integrinβ4 axis promotes cell viability and motility through the activation of phosphorylated AKT (S473) and crosstalk with several documented stemness-related signaling pathways (gray).

Although approximately 70% of breast cancers are ERα positive, and endocrine therapy with agents such as tamoxifen, a selective estrogen receptor modulator [45], and aromatase inhibitors [46], which ablate peripheral estrogen synthesis, can substantially improve disease-free survival, most ERα-positive tumors that initially respond to anti-estrogenic reagents treatment develop resistance to these treatments without any alteration in the ER expression profile [47]. Therefore, estrogen responsive breast cancers progress to a more aggressive form, even if they have a hormonally independent phenotype. Estrogen activates ERα signaling to directly trasnsactivate cell cycle genes and induces ERα-positive breast cancer cell proliferation [48]. And estrogen promotes breast cancer cell invasion and metastasis in lymph nodes and distant organs [49] through direct transactivation of several cell migration genes such as actin-binding protein ezrin [50] and histone deacetylase (HDAC) 6 [51]. Estrogen also promotes the epithelial-mesenchymal transition and stemness of ER-positive breast cancer cells [52, 53] through hedgehog signaling activation [54], G protein-coupled estrogen receptor (GPCER) regulation [55], or crosstalk to Notch signaling [56], SDF-1/CXCR4 signaling [57] and TGFβ signaling [58]. In this study, we showed that estrogen enhances breast cancer cell viability and motility through the ERα-ΔNp63-integrin β4 signaling axis (Fig 6). This novel relationship made a rationale connection between several reported crosstalk of estrogen/ER signaling.

Besides, ΔNp63 also induces epithelial-mesenchymal transition and stemness in both breast cancer tumors-initiating cells and normal mammary stem cells by transactivating several pivotal genes including sonic hedgehog (Shh), GLI family zinc finger 2 (Gli2), and patched1 (Ptch1) genes to activate the hedgehog signaling [26], or Fzd7 Wnt receptor to enhance Wnt signaling [27], or bone morphogenetic proteins (BMP)-4 to promote TGFβ signaling [28]. And ΔNp63 also transcriptionally enhance cell viability [20, 21] and survival [22] through genes involved in signaling pathways that crosstalk with the ERα signaling, such as Shh/Gli and AKT signaling. In addition, ΔNp63 has been reported to mutually inhibit TAp63 [29–31]; however, TAp63 showed only basal-level expression of both mRNA and protein in our results; thus, no estrogen-leading mutually inhibitory effect was observed. TP63 gene is rarely mutant in human cancers [59, 60], which is implying of the epigenetic regulation of TAp63 [61]. However, a detailed understanding of p63 interaction in breast cancer must be elucidated in a future study.

### 2. ERα transactivated ΔNp63 to induce integrin β4/AKT signaling and to enhance breast cancer cell viability and motility

In this study, we introduce an alternative regulation of ΔNp63, which directly regulates the ΔNp63-integrin β4-AKT axis through the activation the estrogen/ERα complex. This novel axis also conferred a rational connection for several findings of previous studies. For instance, loss of integrin β4 inhibits the tumorigenicity and survival ability of ERα-positiveMCF-7 breast cancer cells [11] and ΔNp63 functions as an integrin β4 transcription factor to regulate cellular adhesion and survival [43].

Integrin β4 is an important regulator of both normal breast gland development and breast cancer initiation and progression. Integrin β4 overexpression is also associated with the poor prognosis of breast cancer and several other cancer types [62]. With respect to upstream regulation, integrin β4 is essential for ErbB2-mediated breast carcinogenesis and progression [8] and for P-cadherin-triggered stem cell and invasive properties in basal-like breast cancer cells [63]. With respect to downstream regulation, released integrin β4, which binds directly to laminin, promotes survival and invasion by directly activating PI3K/AKT signaling and RhoA small GTPases [5, 6]. The PI3K/AKT pathway has been well-documented as one of the most frequently dysregulated pathways in cancer, and overactivation of PI3K/AKT signaling pathway results in more aggressive phenotypes of cancer cells [64]. Moreover, overactivation of phosphorylated AKT (pAKT) has been associated with a poor breast cancer prognosis [65]. With respect to crosstalk, integrin β4 cooperates with multiple growth factor receptors to enhance signaling through PI3K, AKT, MAPK, and so on [7] and also aggravates malignant phenotypes.

Besides, estrogen-dependent PI3K activation has also been reported to enhance cell motility and invasion by activating the RhoA/ROCK-2 cascade and ezrin activity in breast cancer cells [50]. These molecules interact with integrin β4 [66], which may explain the regulatory effects of estrogen and ΔNp63 on protruding pseudopodia. In addition, membrane-bound ERs also directly interact with specific domains of kinases, such as c-Src or PI3K, to activate the downstream PI3K/AKT signaling pathway. However, the membrane-bound ERs at the cell surface are thought to initiate non-genomic effects that rapidly alter cell signaling for urgent responses by modulating intracellular signaling cascades, in that, these effects are usually insensitive to mRNA and protein synthesis inhibitors [67, 68].

In conclusion, our results show that estrogen enhances breast cancer cell viability and motility by activatingthe ERα-ΔNp63-integrinβ4 axis and subsequently inducing AKT phosphorylation. However, TAp63 is insensitive to estrogen in ERα-positive MCF-7 breast cancer cells. Conversely, estrogen conferred no inductive effect on this axis and cell behaviors in ERα-negative MDA-MB-231 breast cancer cells. Those findings should be useful for elucidating the downstream crosstalk of estrogen/ER signaling and ΔNp63 signaling. However, it is still not clear whether ERα-ΔNp63-integrin β4 signaling results in tamoxifen-resistance in ERα-positive breast cancer or how integrinβ4 downstream signaling pathways interact during breast cancer progression. Further verification of *in vitro* and *in vivo* molecular regulation and clinical interpretation of breast cancer specimens should be performed in future studies.

## Supporting Information

S1 Fig Estrogen effect on cell viability of MCF-7 and MDA-MB-231 cells. Different concentrations of estrogen (10, 25, 50, and 100nM) were treated to MCF-7 andMDA-MB-231 cells for 4 days, and the cell viability was determined by (A, C) MTT assay and (B, D) trypan blue exclusion assay. Both assays indicated that 10nM estrogen exerted the most effective enhancement for MCF-7 cell viability. Otherwise, higher estrogen concentrations revealed more inhibitory effect on cell viability. But the cell viability of the MDA-MB-231 cells was not modified regardless of the presence or absence of estrogen. Cells treated with 0.1% ethanol were used as thesolvent control group.
(PDF)
