# Supplementary material for: Estrogen Enhances the Cell Viability and Motility of Breast Cancer Cells through the ERα-ΔNp63-Integrin β4 Signaling Pathway
Source: PLoS One. 2016 Feb 4;11(2):e0148301. doi: 10.1371/journal.pone.0148301 (PMC4742232; doi:10.1371/journal.pone.0148301)
Supplement: S1 Fig — Different concentrations of estrogen (10, 25, 50, and 100nM) were treated to MCF-7 andMDA-MB-231 cells for 4 days, and the cell viability was determined by (A, C) MTT assay and (B, D) trypan blue exclusion assay. Both assays indicated that 10nM estrogen exerted the most effective enhancement for MCF-7 cell viability. Otherwise, higher estrogen concentrations revealed more inhibitory effect on cell viability. But the cell viability of the MDA-MB-231 cells was not modified regardless of the presence or absence of estrogen. Cells treated with 0.1% ethanol were used as thesolvent control group. (PDF) [file pone.0148301.s001.pdf]

S1 Fig.

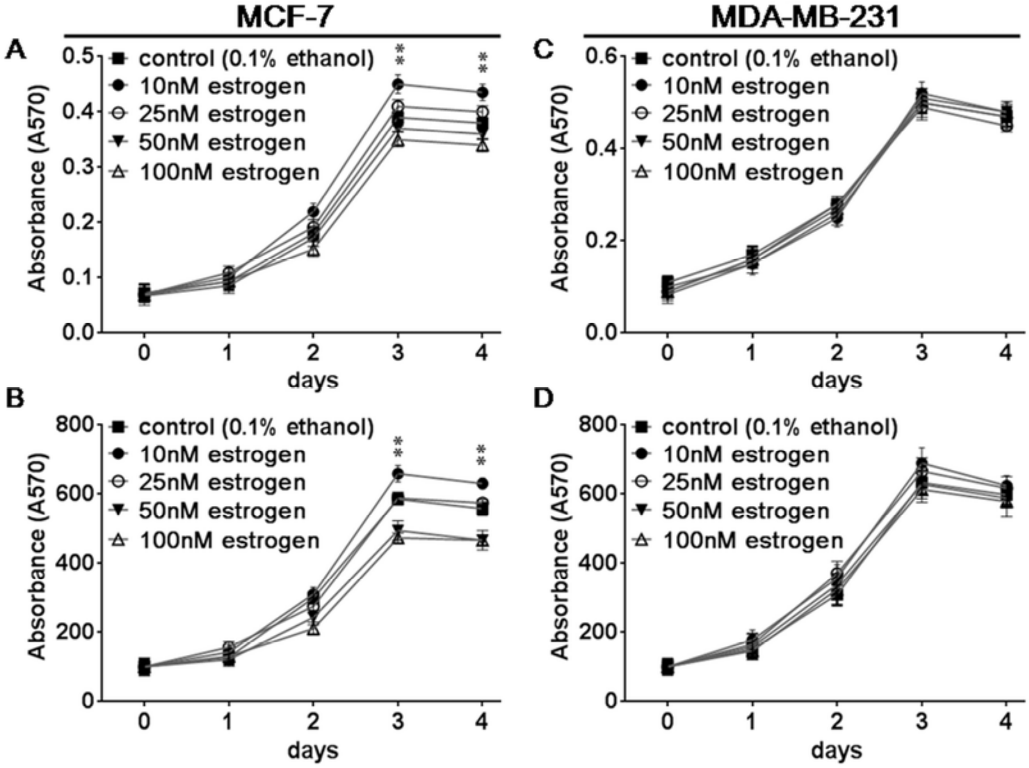

S1 Fig. Estrogen effect on cell viability of MCF-7 and MDA-MB-231 cells

Different concentrations of estrogen (10, 25, 50, 100nM) were treated to MCF-7 and MDA-MB-231 cells for 4 days, and the cell viability was determined by (A, C) MTT assay and (B, D) trypan blue exclusion assay. Both assays indicated that 10nM estrogen exerted the most effective enhancement for MCF-7 cell viability. Otherwise, higher estrogen concentrations revealed more inhibitory effect on cell viability. But the cell viability of the MDA-MB-231 cells was not modified regardless of the presence or absence of estrogen. Cells treated with 0.1% ethanol were used as the solvent control group.
